# ultrasonography of kidney and spleen in clinically healthy llamas and alpacas

**DOI:** 10.1186/s13028-021-00571-5

**Published:** 2021-01-21

**Authors:** Cassandra Eibl, Sonja Franz

**Affiliations:** University Clinic for Ruminants, Department for Farm Animals and Veterinary Public Health, University of Veterinary Medicine Vienna, Veterinärplatz 1, 1210 Vienna, Austria

**Keywords:** Abdominal organs, Kidney, Sonogram, South american camelids, Spleen, Ultrasound, Urinary tract

## Abstract

**Background:**

The ultrasonographic examination technique is a well-established, non-invasive diagnostic tool for diverse conditions in humans and different animal species. The purpose of our study was to describe ultrasonographic localisation, sonographic appearance and dimensions of the kidneys and spleen of clinically healthy llamas and alpacas. Differences between llamas and alpacas and the influence of sex and ages were investigated. Results of this study may aid veterinarians performing ultrasonography in diseased animals and the technique can be used for routine protocol screening.

**Results:**

Ultrasonography was performed in 135 clinically healthy, non-sedated llamas and alpacas. Screening was performed with a 6.6 MHz curve linear transducer with only alcohol as contact medium between the probe and unclipped skin. The kidneys could be imaged from the paralumbar region. The right kidney only was visualized when scanning from the right and the left kidney only from the left. While the left kidney appeared in sagittal view as an oval shape in most llamas and alpacas, in one third of animals the left kidney had a triangular shape. The L-shaped base of the spleen, with its homogeneous, echoic pattern, could be seen craniolateral to the left kidney. Anechoic areas displaying vessels inside the spleen and a thin echoic capsule surrounding the splenic tissue could be differentiated. While sonographic appearances of the examined organs showed no differences between llamas and alpacas, selected dimensions of both of kidney and spleen showed significant differences between species. In terms of age and sex, significant differences in respect of kidney size could be found only in alpacas. Sex seemed to have no influence on kidney and spleen sizes in llamas.

**Conclusions:**

The present study provides species-specific information on ultrasonographic appearance and reference values for kidney and spleen dimensions of clinically healthy llamas and alpacas. Results show differences in organ sizes between llamas and alpacas and in alpacas of different sex and age. The results of this study can be used as references for veterinarians performing ultrasound examinations in diseased animals.

## Background

Ultrasonography is especially helpful as an ancillary test in the clinical work-up of acute and chronic abdominal diseases in camelids, which are reported to be a common cause of death in llamas and alpacas [1, 2]. Ultrasonographic examination of the spleen can be used to identify primary abnormalities and to differentiate strangulating obstructions, such as splenic torsion or displacement, from other causes of abdominal discomfort [3–5].

Common urological diseases of camelids, such as urolithiasis or kidney failure [6–12], further justify the importance of renal ultrasonography for diagnostic purposes and the need for knowledge of their physiological appearance. In urinary tract diseases, ultrasonography provides information about size, position and parenchymal structure of the kidneys and urinary bladder.

However, the routine use of ultrasonography in these species is currently limited by a lack of published physiological findings for kidney and spleen. Descriptions of scanning technique, normal sonographic appearances, including organ size, are rare or even missing [1, 13–15].

Therefore, it was the aim of this study to determine ultrasonographic position, shape, appearance and size of the right and left kidneys and spleen in clinically healthy camelids. Further, it was our goal to clarify if there are species-, age- or sex-specific differences. Data acquired from this study should help veterinarians better interpret ultrasonographic findings in diseased animals.

## Methods

### Animals

This field study was performed in clinically healthy llamas and alpacas of different sex and age from privately owned herds. All animal owners consented to take part at this study by signing a letter of agreement. The animals were chosen for the study at random. Inclusion criteria were that the animals had no pathological findings as revealed by physical examination [16], a body condition score between 2.5 and 3.5 on a point scale [13, 17] and that females, if pregnant, were gestating for less than six months. Animals were divided into two groups, according to age. One group included animals younger than one year and the other included animals one year and older. Species and sex were documented. The study procedures were discussed with owners in advance and approved by the institutional ethics committee of the University of Veterinary Medicine, Vienna, in accordance with Good Scientific Practice guidelines and national legislation.

### Ultrasonographic examination

**Fig. 1:**
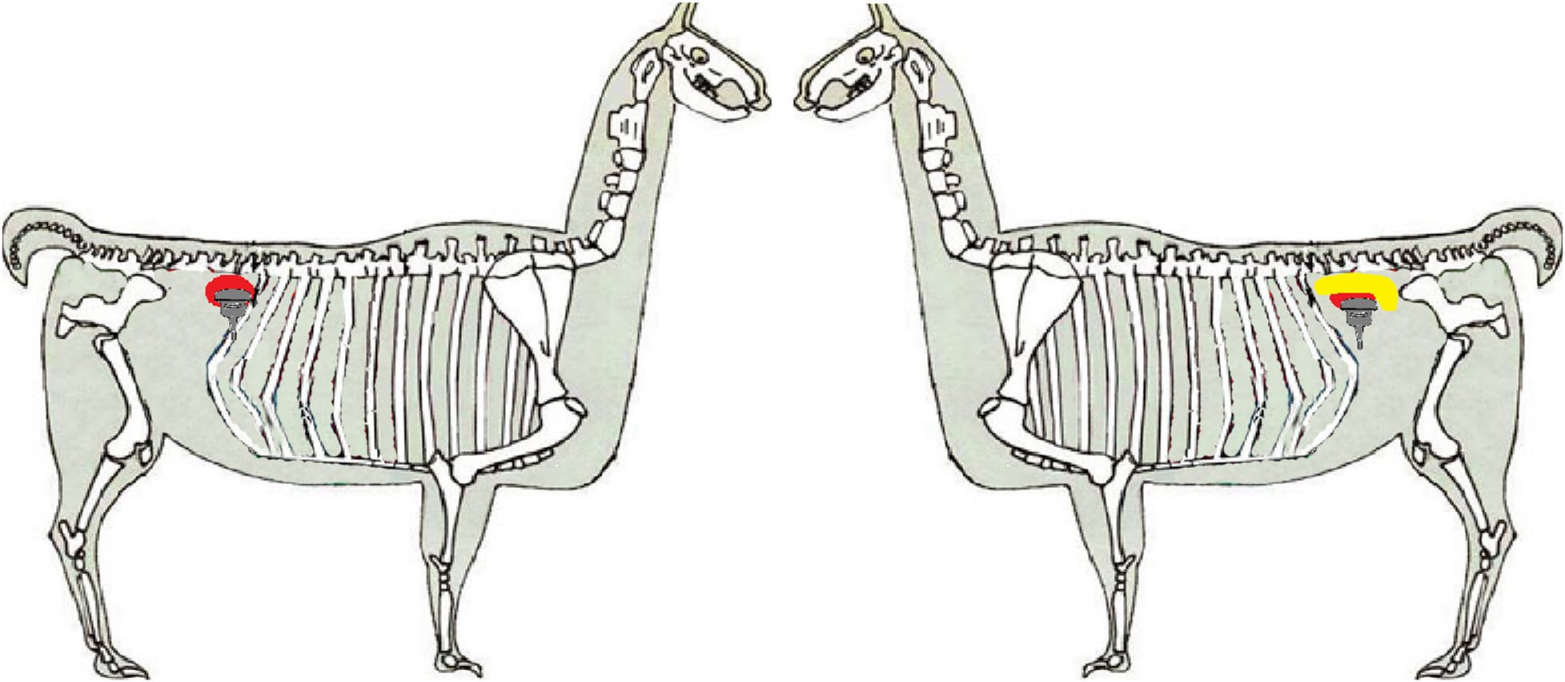
Illustration of the skeleton of a llama. Skeleton of a llama (modified after [33]), location of the transducer for scanning the left kidney (red area) and spleen (yellow area) from the left paralumbar region and the location of the transducer for scanning the right kidney (red area) from the right paralumbar region

The non-sedated animals were restrained by their owners using a halter. Wool fibres in the area of interest were parted with the fingers and solely alcohol (70% ethanol) was used as contact medium between the probe and skin. Ultrasound scanning equipment with a 6.6 MHz curve linear transducer was used (MyLab™One VET, Esaote, Genova, Italy). Ultrasonography was performed over both paralumbar regions (Fig. 1). Each organ was scanned in both sagittal and transverse planes in the same anatomical regions. The sonographic appearance (echogenicity, echoic pattern) of organs, description of probe position in respect of animal numbers, where the organs could be imaged successfully, were also documented (Tables 1 and 2).

**Table 1:** Ultrasonography of kidney and spleen of alpacas (animals ≥ 1 year, n = 96): Results of measurements of selected structures of the scanned organs

Organ	n	Mean (cm)	Min (cm)	Max (cm)	SD
Left kidney—sagittal					
Width	60	4.19	2.77	6.40	0.69
Length	61	7.39	5.61	11.00	0.82
Cortex	30	1.05	0.77	2.46	0.30
Medullary pyramids	30	0.80	0.52	1.61	0.21
Left kidney—transversal					
Width	26	4.98	3.89	6.48	0.65
Cortex	10	0.91	0.64	1.41	0.22
Medullary pyramids	10	0.76	0.50	1.13	0.21
Spleen—sagittal					
Width	20	2.38	1.76	3.32	0.42
Right kidney—sagittalWidth	57	4.00	2.89	5.17	0.52
Length	58	7.10	5.01	9.44	0.78
Cortex	52	0.93	0.61	1.41	0.16
Medullary pyramids	52	0.73	0.42	1.28	0.19
Right kidney—transversal					
Width	31	4.49	3.45	5.82	0.53
Cortex	21	0.88	0.56	1.16	0.14
Medullary pyramids	21	0.65	0.41	0.82	0.11

*n  *number of animals, *Min *minimum, *Max  *maximum, *SD* standard deviation

**Table 2:** Ultrasonography of kidneys and spleens of llamas (n = 32): Results of measurements of selected structures of the scanned organs

Organ	n	Mean (cm)	Min (cm)	Max (cm)	SD
Left kidney—sagittal					
Width	26	5.16	3.76	7.17	0.90
Length	26	8.72	6.46	12.04	1.40
Cortex	12	1.12	0.74	2.33	0.42
Medullary pyramids	12	0.81	0.47	1.17	0.19
Left kidney—transversal					
Width	10	5.79	4.18	7.10	0.93
Cortex	5	1.11	0.62	1.48	0.39
Medullary pyramids	5	0.89	0.44	1.47	0.37
Spleen—sagittal					
Width	13	2.95	2.15	5.63	0.92
Right kidney—sagittal					
Width	20	4.84	3.47	6.40	0.83
Length	20	8.34	7.12	9.72	0.78
Cortex	14	1.07	0.70	1.87	0.28
Medullary pyramids	13	0.76	0.52	0.95	0.17
Right kidney—transversal					
Width	13	5.71	4.15	6.84	0.76
Cortex	12	1.12	0.72	2.12	0.18
Medullary pyramids	10	0.85	0.54	1.21	0.18

*n* number of animals, *Min*  minimum, *Max* maximum, *SD* standard deviation

### Ultrasonographic measurements

For determination of organ sizes, single measurements of selected parameters were performed on stored sonographic images using electronic callipers and MyLab_Desk Software (Esaote). For determination of the size of both kidneys, the distance between cranial and caudal poles (length) and the distance between medial and lateral margins (width) were measured in the sagittal scanning view through the medullary pyramid region. Distances between lateral and medial margins in the transversal view at the level of the renal hilus were also measured. In addition, the distance between the renal cortex and medullary pyramids were measured in both sagittal and transverse planes. The width of the spleen was measured in the sagittal scanning view between lateral and medial margins before its visible physiological bend, at the level of the caudal pole of the kidney.

### Statistical analysis

The technical variability of the measuring instrument was tested at the beginning of the study by estimation of the variation coefficient. There was no relevant effect on measurements. The descriptive statistic was performed using IBM SPSS ver. 2018. The data are presented as means ± standard deviation (SD) and the Kolmogorov–Smirnov test was used to test data for normal distribution. Measurements were compared between the two species (llama and alpaca) and the measurements were analysed for differences influenced by age (group 1: animals < one year, group 2: animals ≥ one year; the values of group 1 are only calculated in the age comparison) and sex (male / female) by means of Student’s t-test using a significance level of 5% (*P* < 0.05) and a confidence interval of 95%.

## Results

Ultrasonographic examination of kidneys and spleens was performed in 135 animals, mainly alpacas (n = 103) and a smaller proportion of llamas (n = 32). There were 99 females (76 alpacas, 23 llamas) and 36 males (27 alpacas, 9 llamas). 29 alpacas were pregnant. Only seven alpacas were younger than one year and 128 camelids (96 alpacas, 32 llamas) were one year or older. The youngest animal was four months old and the oldest was 17 years old (alpaca < one year: mean: 5.3 months, SD ± 1.2; alpaca > one year: 5.9 ± 3.6; llama > one year: 6.6 ± 4.2).

Tables 1 and 2 show the number of animals older than one year in which kidney and spleen could be imaged successfully. The differences are generated due to animals showing defence reactions and adverse behaviour. Poor image quality was responsible for exclusion of five animals (three male and two female alpacas).

### Ultrasonography of the left kidney

**Fig. 2:**
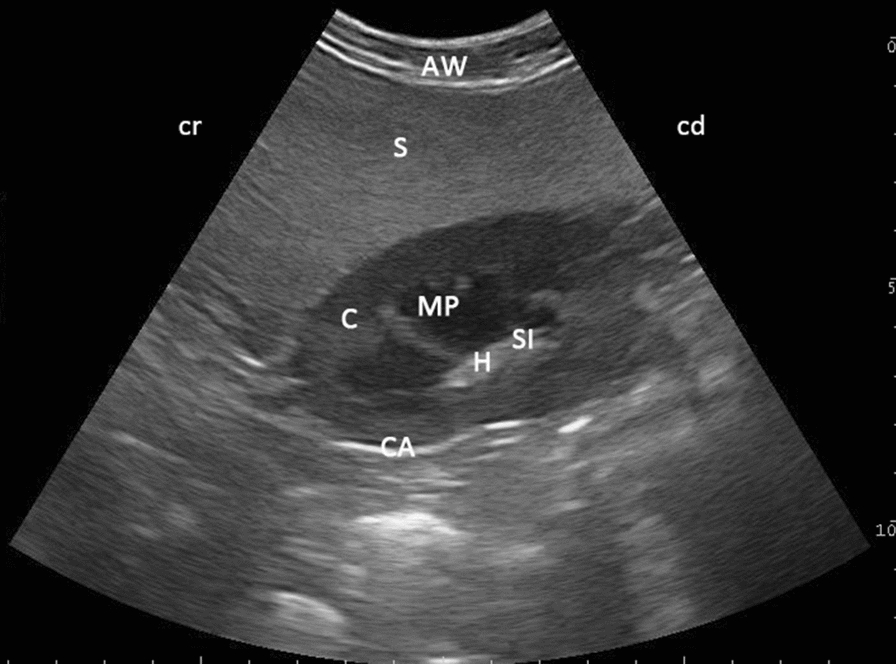
Ultrasonogram of the oval shape of the left kidney in sagittal view in a five-year-old llama. Note the echoic cortex (C), anechoic medullary pyramids (MP) and the hyperechoic renal sinus (SI) and renal hilus (H), as viewed through the left paralumbar fossa using a 6.6 MHz probe. CA: capsule, AW: abdominal wall, S: spleen, cr: cranial, cd: caudal

The left kidney was best imaged from the left paralumbar region when the probe was positioned at the level of the 4th to 7th transverse processes of the lumbar spine, directly ventrally to the bony structures (Fig. 2). Several renal structures could be visualised sonographically in this area in both sagittal and transverse views. Kidneys in all animals were smooth in outline and surrounded by a thin hyperechoic line, corresponding to the renal capsule. The normal renal cortex was uniformly homogenously echoic, slightly hypoechoic when compared with the splenic parenchyma. The medullary pyramids were adjacent to the renal cortex and appeared as circular hypoechoic structures. The renal sinus was displayed as a hyperechoic thin band. While the renal hilus was hyperechoic, the renal arteries, veins and ureters could not be differentiated without Doppler ultrasonography. Ureters were not visible in any animal, whether viewed in transverse or sagittal planes.

**Fig. 3:**
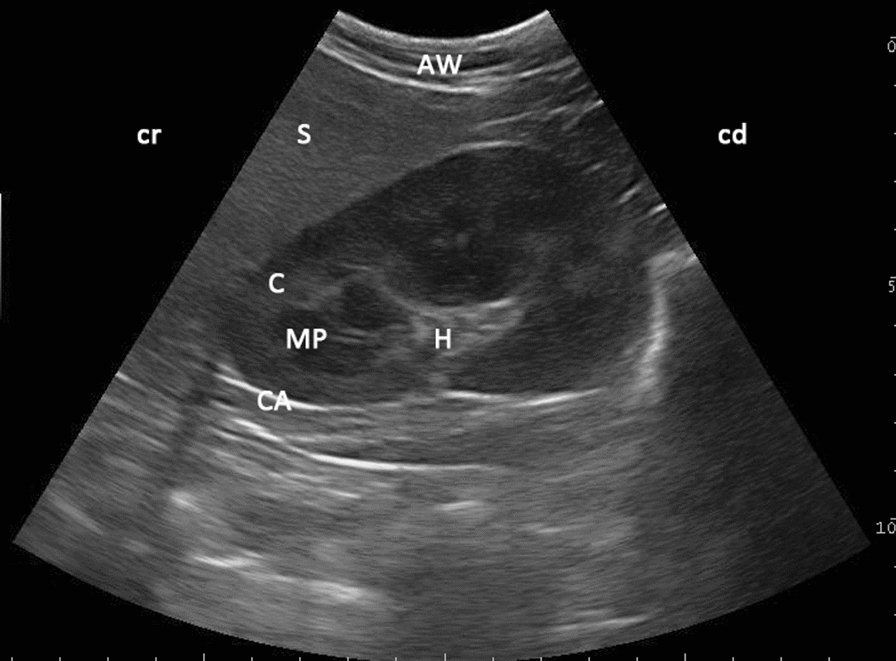
Ultrasonogram of the triangular shape of the left kidney in sagittal view in a four-year-old alpaca. The shape of the left kidney of an alpaca with the echoic cortex (C) and anechoic medullary pyramids (MP) as viewed from the left paralumbar fossa using a 6.6 MHz probe; *H* hilus, *CA* capsule, *AW* abdominal wall, *S* spleen, *cr* cranial, *cd* caudal

**Fig. 4:**
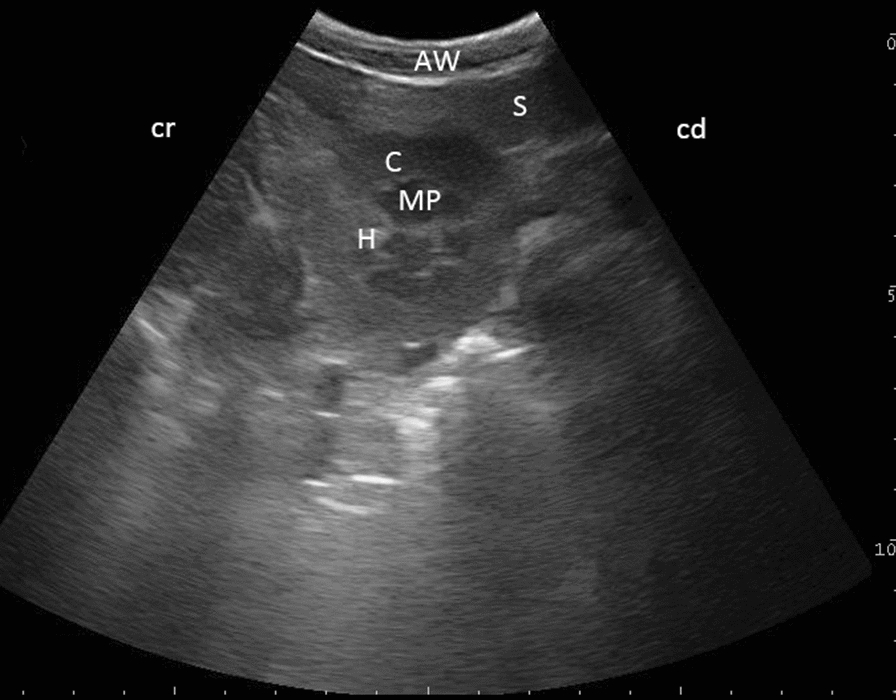
Ultrasonogram of the left kidney in transverse view in a six-year-old alpaca. Note the “euro-sign” shape of the medullary pyramids (MP) due to the precise medial scan at the level of the hilus (H), as viewed from the left paralumbar fossa using a 6.6 MHz probe. The distance between lateral and medial margins and between the renal cortex and medullary pyramids were measured. *C* cortex, *AW* abdominal wall, *S* spleen, *cr* cranial, *cd* caudal

When the probe was positioned for a sagittal view, the kidney appeared oval (Fig. 2) with a smooth margin. However, in 30 (27.3%) out of 92 animals, regardless of species, the left kidney appeared more triangular in shape (Fig. 3). By positioning the probe in the transverse position, the kidney appeared as an oval-to-circular structure (Fig. 4) in all animals. When scanned at the level of the hilus (Fig. 4), the medullary pyramids appeared as “inverted Euro sign” shapes.

Sagittally, the length of the left kidney could be measured in 92 (68.1%) animals (llamas and alpacas, both age groups), the width of the left kidney in 90 (66.7%) animals and the thickness of both the renal cortex and the medullary pyramids in 45 (33.3%) animals. In the transverse view, the width of the left kidney could be measured in 38 (28.1%) animals and both the renal cortex and the medullary pyramids could be measured in 17 (12.6%) animals. Tables 1 and 2 display organ measurements for both adult species (animals ≥ 1 year), indicating the size of selected structures of the left kidney.

### Ultrasonography of the right kidney

The right kidney was best viewed sonographically by positioning the probe in the right paralumbar fossa immediately caudal to the last rib, ventral to the lumbar vertebrae (Fig. 1). In comparison to sonographic visualization of the left kidney, the right kidney was positioned slightly more cranially.

**Fig. 5:**
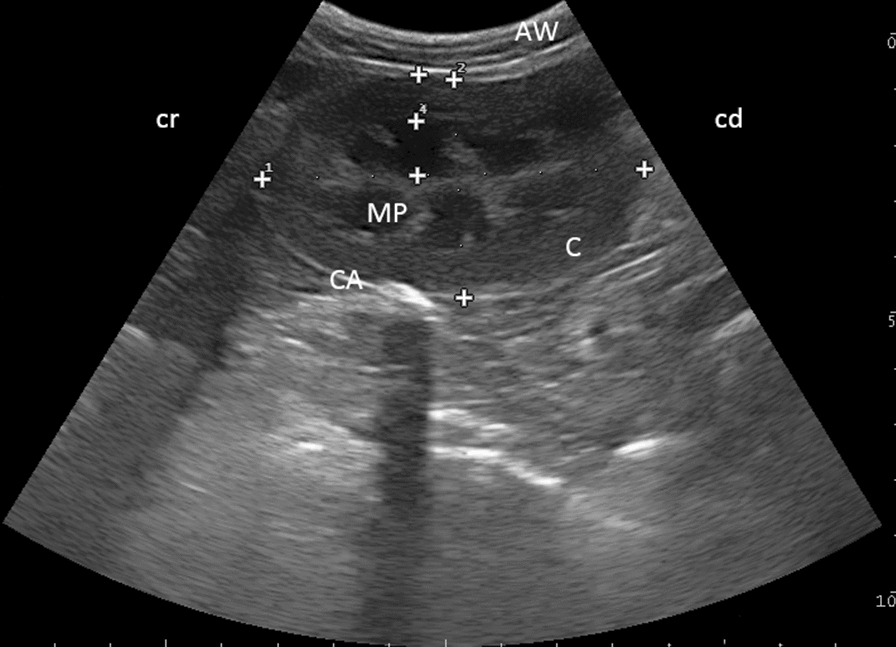
Ultrasonogram of the right kidney in a four-year-old alpaca using a 6.6 MHz probe. Sagittal view through the region of the medullary pyramids as scanned from the right paralumbar region. The right kidney of an alpaca with the cortex (C), the medullary pyramids (MP) and the hyperechoic capsule (CA), as viewed from the right paralumbar fossa. Measurements of the length and width of the kidney and the width of the cortex and medullary pyramids are shown. *AW* abdominal wall, *cr* cranial, *cd* caudal

In 92 (68.1%) animals, the right kidney was imaged as an oval shape in the sagittal view and as an oval-to-circular shape in the transverse view. Sonographically, no differences were evident between kidneys. Measurements of selected renal structures were performed while positioning the probe in the sagittal scanning view through the region of the medullary pyramids (Fig. 5) and transverse at the level of the hilus in the right paralumbar region. In the sagittal view, the length of the right kidney could be measured in 83 (61.5%) animals (both age groups), the width in 82 (60.7%) animals, the thickness of renal cortex in 71 (52.6%) and the thickness of the medullary pyramids in 70 (51.9%) animals. In the transverse view, the width of the right kidney could be measured in 48 (35.6%) animals, the thickness of the renal cortex in 36 (26.7%) and the thickness of the medullary pyramids in 33 (24.4%) animals. Tables 1 and 2 display organ measurements for both adult species (animals ≥ 1 year), indicating the size of selected structures of the right kidney.

### Ultrasonography of the spleen

**Fig. 6:**
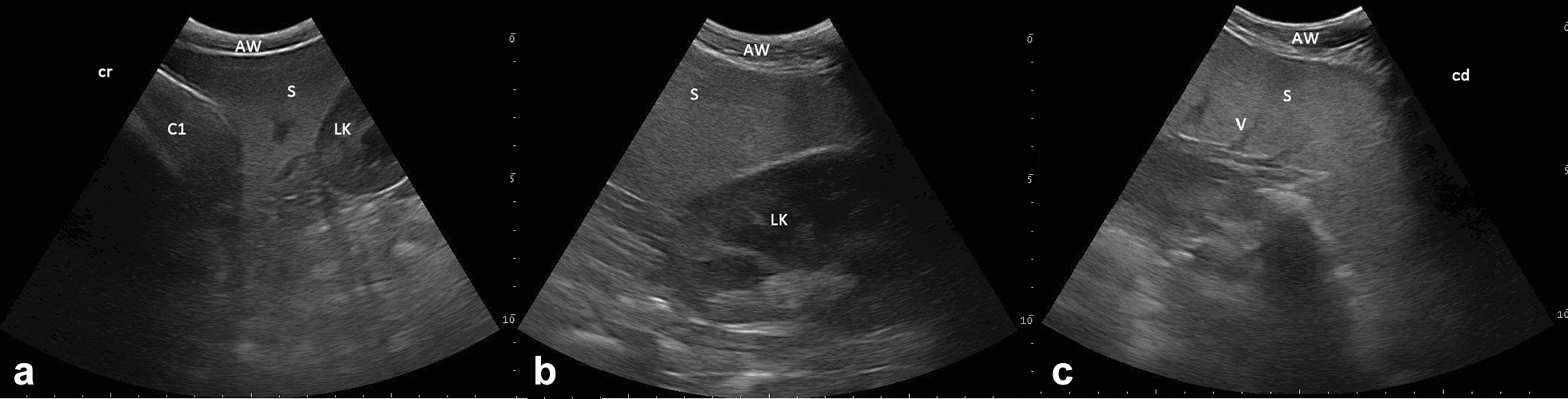
Sonographic appearance of the spleen in sagittal view in a 6 year-old alpaca. **a** The spleen (S), as viewed from the left paralumbar fossa, is caudal to the wall of compartment 1 (C1) and **b** craniolateral to the left kidney (LK). **c** The bend of the splenic tissue in the medial direction. *AW* abdominal wall, *V* vessel, *cr* cranial, *cd* caudal, 6.6 MHz

**Fig. 7:**
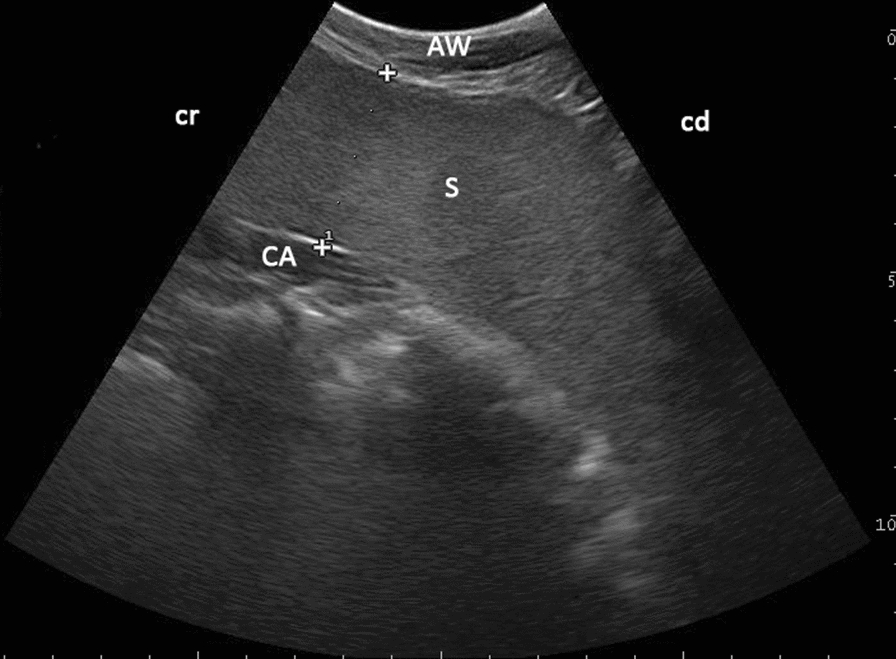
Ultrasonographic appearance of the spleen in sagittal view in an eight-year-old llama. The bend of the llama spleen (S) as viewed from the left paralumbar fossa using a 6.6 MHz probe. Note the homogeneous echoic parenchyma and the capsule (CA) surrounding the spleen as a thin, hyperechoic line. The width of the spleen was measured between the lateral and medial margins. *AW* abdominal wall, *cr* cranial, *cd* caudal, 6.6 MHz

The base of the spleen, with its homogeneous and echoic pattern, was visualized by positioning the probe cranial to the left kidney in the left paralumbar fossa at the level of the 3^th^ to 7^th^ transverse processes of the lumbar spine (Fig. 1). In 35 (25.9%) of 135 animals (both age groups), the spleen could be examined sonographically (Tables 1 and 2). In all camelids examined, the spleen was L-shaped when the probe was positioned sagittally. The spleen was located caudal to compartment 1 and craniolateral to the left kidney (Fig. 6), bending in a medial direction at the level of the caudal pole of the left kidney. When visualising the spleen in sagittal view, it was found best to freeze the image on the monitor to determine the distance between the splenic parenchyma and the visible physiological bend (Fig. 7). Anechoic vessels in the homogeneous echoic splenic parenchyma and a thin hyperechoic line surrounding the spleen, corresponding to the capsule, could be visualised.

### Statistical analysis of ultrasonographic measurement

The measurements of selected renal and splenic structures were compared by species, sex and age (Tables 3, 4, 5). 135 animals were examined, of which only seven alpacas were younger than one year. Therefore, the influence of species and sex was only evaluated in adult alpacas and llamas. There were significant differences between species in the measurements of the length of the left kidney in sagittal view, the width of the renal cortex of the left kidney in transversal view, and the width of the right kidney in sagittal view. However, there was no significant difference concerning widths of medullary pyramids (Table 3). Sex had no significant influence on measurements of kidneys and spleens in the llama group. Only three measurements (width and length of the right kidney in the sagittal view, width of renal cortex of the right kidney in transversal view) were significantly different in female and male alpacas (Table 4). Although there were only seven alpacas younger than one year, the influence of age was evaluated in the alpaca group. However, there were several significant differences in measurements with respect to the left kidney (width and length in sagittal view, width in transverse view), and the right kidney (width and length in sagittal view, width and width of the cortex in transverse view) in the alpaca group (Table 5).

**Table 3:** Significant differences in measurements of several structures of the scanned organs comparing llamas and alpacas (animals ≥ 1 year)

Organ	*P*	95% CI of differences	
		Lower limit	Upper limit
Left kidney—sagittal			
Width	0.061	− 1.31	− 0.60
Length	0.001*	− 1,80	− 0.84
Cortex	0.274	− 0.31	0.16
Medullary pyramids	0.994	− 0.15	0.13
Left kidney—transversal			
Width	0.282	− 1.35	− 0.24
Cortex	0.045*	− 0.53	0.14
Medullary pyramids	0.514	− 0.44	0.19
Spleen—sagittal			
Width	0.123	− 1.04	− 0.08
Right kidney—sagittal			
Width	0.007*	− 1.15	− 0.51
Length	0.380	− 1.63	− 0.82
Cortex	0.096	− 0.26	− 0.02
Medullary pyramids	0.673	− 0.14	0.08
Right kidney—transversal			
Width	0.254	− 1.62	− 0.81
Cortex	0.081	− 0.41	− 0.06
Medullary pyramids	0.193	− 0.31	− 0.08

*Significant values, *CI* confidence interval

**Table 4:** Significant differences in measurements of several structures of the scanned organs in the alpaca group (animals ≥ 1 year) influenced by sex

Organ	*P*	95% CI of differences	
		Lower limit	Upper limit
Left kidney—sagittal			
Width	0.933	− 0.16	0.72
Length	0.089	− 0.06	0.94
Cortex	0.548	− 0.21	0.39
Medullary pyramids	0.335	− 0.11	0.32
Left kidney—transversal			
Width	0.471	− 1.35	0.64
Cortex	0.887	− 0.53	0.61
Medullary pyramids	0.391	− 0.32	0.73
Spleen—sagittal			
Width	0.663	− 0.56	0.36
Right kidney—sagittal			
Width	0.022*	0.05	0.64
Length	0.000*	0.44	1.25
Cortex	0.287	− 0.05	0.16
Medullary pyramids	0.230	− 0.05	0.20
Right kidney—transversal			
Width	0.775	− 0.39	0.51
Cortex	0.036*	− 0.01	0.31
Medullary pyramids	0.682	− 0.11	0.16

*Significant values, *CI* confidence interval

**Table 5:** Significant differences in measurements of several structures of the scanned organs in the alpaca group (animals < one year and animals ≥ 1 year) influenced by age

Organ	*P*	95% CI of differences	
		Lower limit	Upper limit
Left kidney—sagittal			
Width	0.021*	− 1.54	− 0.13
Length	0.000*	− 2.56	− 1.05
Cortex	0.143	− 0.63	0.09
Medullary pyramids	0.261	− 0.42	0.12
Left kidney—transversal			
Width	0.004*	− 2.48	− 0.53
Cortex	0.249	− 0.57	0.17
Medullary pyramids	0.307	− 0.52	0.18
Spleen—sagittal			
Width	0.281	− 0.99	0.30
Right kidney—sagittal			
Width	0.001*	− 1.39	− 0.39
Length	0.001*	− 2.08	− 0.61
Cortex	0.660	− 0.19	0.12
Medullary pyramids	0.191	− 0.31	0.06
Right kidney—transversal			
Width	0.001*	− 1.56	− 0.45
Cortex	0.014*	− 0.43	− 0.05
Medullary pyramids	0.414	− 0.13	0.29

*Significant values, *CI* confidence interval

## Discussion

The purpose of this study was to describe an appropriate scanning procedure and identify possible physiologic variations in the sonographic appearances of kidneys and spleens in healthy llamas and alpacas. Ultrasonography as a complementary diagnostic modality in diseased camelids has become very popular and is a helpful additional diagnostic tool to physical examination and laboratory tests [18–24]. However, descriptions of normal sonographic appearances of different organs in healthy camelids and the scanning technique itself is rarely found in existing literature. In most of these studies, only a low number of animals were used and the animals were sedated [8, 14].

We focused on performing ultrasonography under field conditions without sedation and attempted to examine a higher number of animals. The latter was necessary to establish reference values for the sizes of the scanned organs for the first time that can be used to compare diseased animals. However, by selecting preferentially for numbers, it was not feasible simultaneously to perform laboratory tests such as haematology and serum biochemical analyses) to look for possible correlations between abnormalities not detectable by physical examination alone. It cannot be excluded that some animals may have been experiencing relevant sub-clinical diseases that might have influenced the appearance and sonographic measurements of the scanned organs. But this possibility is unlikely as the variability of the measured values show, unless all animals were affected.

Wool fibres were not clipped for ultrasonographic examination. The fibres were only parted with the fingers and only alcohol was used as the contact medium between probe and skin. This technique proved to be useful as the wool only seriously interfered with interpretation of the sonogram in five animals. This finding is important, as the fleece is of great economic value to the owner and shearing for routine ultrasonography can be undesirable.

Scanning was not successful in some animals due to lack of cooperation or adverse behaviour and when the procedure took too much time. In healthy camelids this is not surprising because of the nature of their normal behaviour. They usually move away from strangers, especially when physical contact is attempted in their abdominal region.

A curve linear probe with the frequency of 6.6 MHz was used for the examinations, which turned out to be feasible for percutaneous scanning. This is of great advantage, since many ultrasound devices that are used in practice are equipped with similar frequencies. Both kidneys were best viewed from the paralumbar regions. Renal structures such as the capsule, the renal cortex, the medullary pyramids and the renal hilus could all be differentiated. However, in all animals examined the normal non-dilated ureter could not be viewed sonographically. This is also true for cattle, rams and goats [25–28]. The various structures of the kidneys reported had the same sonographic appearances, whereby the following structures could be imaged: a hyperechoic capsule, a hypoechoic renal cortex, anechoic medullary pyramids and the hyperechoic sinus and hilus.

In camelids the left kidney only could be viewed via the left abdominal approach and the right kidney via the right abdominal approach. It was evident that the right kidney was best imaged when placing the probe more cranially in the right paralumbar region in comparison to placement of the probe for visualisation of the left kidney. The more caudal location of the left kidney could be due to its location next to the spleen.

In contrast to llamas and alpacas, in most ruminants both kidneys are visible from the right flank when using the percutaneous technique. This is due to the rumen that fills almost the entire left half of the abdomen [25–30]. Hence it is surprising that the camelid C1, that is comparable to the rumen of cattle and small ruminants, does not displace the left kidney medially and it could only be imaged in our study from the left abdomen.

When scanning the left kidney, it was evident that in almost one third of the camelids the left kidney had a different shape, a triangular shape, in the sagittal view compared to other animals, that had an oval-shaped kidney. The reason for that is unknown, and could be due to close proximity to the spleen during embryonic development, or even due to undetected pathological change of the kidney.

The ultrasonographic measurements of some renal structures were significantly different between alpacas and llamas, such that the llama kidney is larger than that of the alpaca. The main reason could be the difference in body sizes between the species, as llamas usually have a higher body weight and are taller. As this project was a field study, no body weight data was available. Therefore, it was not possible to test for a correlation between ultrasonographic organ size and body weight. Width and length measurements of both kidneys in the alpaca group were significantly different, depending on age, and varied by sex in merely single parameters. It is of course possible that male and older animals possess larger organs compared to female and younger animals. Nevertheless, measurement results must be interpreted with caution due to the relatively low number of animals represented. The low number of animals investigated was also responsible for the lack of statistical significance between measurements in the llama group.

Sonographically performed measurements of the kidney are also available for goats and rams. The reported length of the kidneys of goats is between 6.6 and 9.9 cm and the width is between 3.9 and 6.3 cm. In rams, kidney size varies from 7.5 to 9.1 cm in length and 4.1 to 5.4 cm in width [25–27]. Comparing these dimensions between camelids and small ruminants, llamas and alpacas appear to have kidneys of similar width. This is surprising, since adult alpacas, and lamas in particular, have significantly more body weight than goats and rams. This illustrates that larger animals do not necessarily have correspondingly larger internal organs. There may be microscopic structural differences that account for species-specific physiological functions while the physical size remains apparently constant. Further studies have to be performed in order to look for the influence of breed or conformation on the dimensions of these organs.

The spleen was best viewed in the left paralumbar fossa at the level of the 3rd to 7th transverse processes of the lumbar spine, in both transverse and sagittal views. For anatomic reasons this is different to cattle and small ruminants, where the spleen can be viewed between the 8th and the 12th intercostal spaces [26, 31, 32]. Knowing the ultrasonographic L-shape of this organ and its location next to the kidney is important for performing and interpreting these sonograms. The ultrasonographic appearance of the spleen is similar to that in cattle and small ruminants. The spleen is surrounded by an echoic capsule that can be clearly differentiated. The splenic parenchyma consists of multiple, small, regularly spaced homogeneous echoes, and the splenic vessels appear anechoic [26, 31]. Measurement of the distance between medial and lateral margins of the splenic parenchyma before the splenic bend became visible proved to be a suitable location, since it was reproducible. However, results of measurements cannot be directly compared with other animal species since the sites of measurement vary. There was no difference in sonographic appearances between alpaca and llama. The width of the spleen was significantly different, such that the llama spleen was bigger. The reason could simply reflect the difference in body sizes.

## Conclusions

Percutaneous ultrasonography of the kidneys and spleens of llamas and alpacas is a feasible technique. It can be performed without clipping wool fibres in the region of interest, using only alcohol as contact medium between probe and skin. Reference values for dimensions of ultrasonographically examined kidneys and spleens of healthy animals are presented. Typical sonograms reveal the oval, and in some cases even triangular, shape of the left kidney and the typical L-shape of the spleen. Differences in physiological organ size between llamas and alpacas and between alpacas of different sex and age must be taken into account when performing ultrasonography in camelids.

## Data Availability

All data generated or analyzed during this study are included in this published article.
